# Suppression of Spotted Wing Drosophila, *Drosophila suzukii* (Matsumura), in Raspberry Using the Sterile Insect Technique

**DOI:** 10.3390/insects16080791

**Published:** 2025-07-31

**Authors:** Sebastian Hemer, Zeus Mateos-Fierro, Benjamin Brough, Greg Deakin, Robert Moar, Jessica P. Carvalho, Sophie Randall, Adrian Harris, Jimmy Klick, Michael P. Seagraves, Glen Slade, Michelle T. Fountain, Rafael A. Homem

**Affiliations:** 1 BigSis, 7-9 Portman Centre, 37-45 Loverock Road, Reading RG30 1DZ, UK; zmateosf@purdue.edu (Z.M.-F.); rob@bigsis.tech (R.M.); jessica@bigsis.tech (J.P.C.); sophie@bigsis.tech (S.R.); glen@bigsis.tech (G.S.); rahomem@gmail.com (R.A.H.); 2 Department of Entomology, Purdue University, 901 Mitch Daniels Blvd., West Lafayette, IN 47907, USA; 3 Niab East Malling, New Road, East Malling, Kent ME19 6BJ, UK; benbrough1999@gmail.com (B.B.); greg.deakin@niab.com (G.D.); adrian.l.harris@niab.com (A.H.); michelle.fountain@niab.com (M.T.F.); 4 Driscoll’s Inc., 3391 E. Hueneme Rd., Oxnard, CA 93033, USA; jimmy.click@driscolls.com (J.K.); michael.seagraves@driscolls.com (M.P.S.)

**Keywords:** biodiversity, mark–release–recapture, soft fruit, SWD, wind tunnel

## Abstract

*Drosophila suzukii* is an invasive pest of many fruit crops worldwide. Employing the Sterile Insect Technique (SIT) could mitigate *D. suzukii* population growth and crop damage. This study evaluated the efficacy of SIT on commercial fruit, by (1) validating the quality of irradiated sterile males (male mating competitiveness, courtship, and flight performance) in the laboratory, and (2) assessing population suppression and fruit damage reduction in commercial raspberry fields. Treatment with SIT was compared to the grower’s standard chemical insecticide program throughout the season. The principal metrics of efficacy were trap counts of wild adult female *D. suzukii* in crops and larvae per fruit during harvesting. These metrics together with monitoring of border areas allowed targeting of high-pressure areas with higher releases of sterile males, to maximise efficacy for a given release number. The sterile male *D. suzukii* were as competitive as their fertile non-irradiated counterparts in laboratory mating competitiveness and flight performance studies while fertility egg-to-pupae recovery was reduced by 99%. In commercial raspberry crops, season-long releases of sterile males significantly suppressed the wild *D. suzukii* population, compared to the grower standard control strategy; with up to 89% reduction in wild female *D. suzukii* and 80% decrease in numbers of larvae per harvested fruit. Additionally, relative fruit waste (i.e., percentage of harvested fruits rejected for sale) at harvest was reduced for early, mid and late harvest crops, by up to 58% compared to the grower standard control. SIT has the potential to provide an effective and sustainable strategy for managing *D. suzukii* in raspberries, increasing marketable yield by reducing adult populations, fruit damage and waste fruit. SIT could therefore serve as a valuable tool for integrated pest management practices in berry production systems.

## Simple Summary

*Drosophila suzukii*, an invasive fruit pest, poses significant challenges to global soft fruit production. This study evaluated the efficacy of the Sterile Insect Technique (SIT) as a potential management strategy for *D. suzukii* in commercial raspberry fields. Laboratory experiments assessed the quality of irradiated sterile males, while field trials compared SIT to standard chemical insecticide protocols. Results showed that sterile males were equally competitive as their fertile counterparts in mating and flight performance, with 99% reduced egg-to-pupae recovery. In commercial raspberry crops, season-long releases of sterile males significantly suppressed wild *D. suzukii* populations, reducing numbers of wild females by up to 89% and larval infestation in harvested fruit by 80%. Furthermore, the implementation of SIT reduced relative fruit waste during harvest by up to 58% compared to standard control methods. These findings demonstrate that SIT has the potential to provide an effective and sustainable strategy for managing *D. suzukii* in raspberries, reducing waste fruit and serving as a valuable tool for integrated pest management in berry production systems.

## 1. Introduction

The Sterile Insect Technique (SIT) has been successfully used for over 70 years to control many pests worldwide including the new world screwworm fly (*Cochliomyia hominivorax*) [1], Mediterranean fruit fly (*Ceratitis capitata*) [2], Mexican fruit fly (*Anastrepha ludens*) [3], and codling moth (*Cydia pomonella*) [4]. SIT is highly effective and more environmentally friendly than most other pest management approaches (e.g., chemical insecticides) because it is species-specific and non-toxic with minimal environmental impact [5,6].

SIT involves the mass production and sterilisation of male target insects, which are released regularly into a treated area to mate with wild females which then have no or fewer offspring [5]. SIT is ideal as a preventative tool, since the released sterile males will instinctively seek out wild females even at low pest populations, when it is also more efficient to outnumber their wild counterparts. A more widespread adoption of SIT as the primary pest control method for sexually reproducible insect pests has been impeded primarily by the prohibitive costs associated with the large-scale production of sterile males [3].

The relatively recent expansion of the geographic range of *Drosophila suzukii*, commonly known as the spotted wing drosophila, has significantly impacted fruit crops worldwide [7,8,9]. This pest causes huge economic losses in a wide range of soft- and stone- fruits, including berries (e.g., raspberries; *Rubus idaeus*) [10] and cherries (e.g., sweet cherries; *Prunus avium*) [11]. Unlike most other Drosophila species, *D. suzukii* possesses a serrated ovipositor to pierce the epicarp of a ripening fruit from where larvae feed, making the fruit more susceptible to fungal infection and other insect damage leading to accelerated deterioration in fruit quality [12,13]. The latter leads to higher production costs, reduced yield and increased waste [14,15]. As such, effective management strategies are crucial to mitigate the detrimental effects of *D. suzukii* infestation on fruit production [16].

Despite being the most common management technique, the use of chemical insecticides to control *D. suzukii* is highly undesirable, leaving residues on fruit and disrupting beneficial insects such as predators, parasitoids and pollinators, which are relied upon in fruit production for pest control and pollination [16]. Furthermore, since *D. suzukii* is present in multiple life stages (i.e., eggs, larvae and adults) at the same time in and around the crop, chemical insecticides have limited efficacy, typically killing only adults within the crop whereas eggs and larvae inside the fruits remain largely unaffected. This leaves the crop area susceptible to rapid reinvasion from the surrounding habitat and from development of early life stages inside the crop. Insecticide application pre-harvest safety intervals further constrains their usefulness.

Cultural management practices that remove *D. suzukii* resources from the environment such as unmarketable fruit, dropped fruit and other hosts such as wild blackberries (*Rubus* spp.) are costly and sometimes impossible. For example, wild blackberries and wild sweet cherries, which are primary hosts for *D. suzukii*, are widespread in the UK, and commonly found in hedges and woodlands, areas where pesticide use is restricted. The presence of these hosts in pesticide-restricted areas complicates pest management strategies, as *D. suzukii* can multiply and spread from these semi-natural habitats into cultivated crops [10,17].

One of the main advantages of SIT over chemical control strategies, is that sterile males can be released into, and disperse into crop borders or neighbouring woodlands, thereby containing wild populations adjacent to crop area which would otherwise be a source of invasion. In addition, *D. suzukii* sterilised through irradiation [18,19] is more likely to be approved by authorities compared to sterilisation through genetic modification [20,21,22,23], especially in Europe.

Gard et al. [24] did not find a decrease in the proportion of infested strawberries in a cage study using two different release ratios of sterile to fertile bisexual insects (5:1 and 1:1). In contrast to this, we have previously shown that SIT, based on male-only releases at higher sterile-to-wild male ratios, is effective in controlling *D. suzukii* in commercial strawberries grown under open polytunnels [18]. Despite being one of the main crops affected by *D. suzukii*, strawberry is usually less vulnerable to *D. suzukii* damage compared to other crops. Raspberries, for instance, have the highest host potential index among seven tested *D. suzukii* hosts—strawberries (*Fragaria × ananassa*), raspberries, blackberries, blueberries (*Vaccinium corymbosum*), sweet cherries, table grapes (*Vitis vinifera*) and peaches (*Prunus persica*) [25]. The relative host attractiveness to *D. suzukii* has recently been linked to the prevalence of Saccharomycetales yeasts [26].

The aim of this study was to evaluate the efficacy of SIT to control *D. suzukii* in raspberry. Specifically, we evaluated the efficacy of SIT on commercially produced raspberries, by (1) validating the quality of irradiated sterile male *D. suzukii* for mating competitiveness and dispersal, and (2) quantifying *D. suzukii* population suppression and fruit damage in SIT treated commercial raspberry fields.

## 2. Materials and Methods

### 2.1. Quality of Irradiated Sterile Male D. suzukii

*Drosophila suzukii* used in all laboratory experiments (in 2022) and the field trial (in 2023; Section 2.3) were obtained from a laboratory culture originating from adults collected in and around strawberry fields in Kent, UK in 2021. *Drosophila suzukii* were cultured on a standard *D. melanogaster* cornmeal diet (adapted from the Bloomington Stock Centre recipe) (distilled water 1 L, agar 10 g, table sugar 90 g, pre-cooked maize 90 g, baker’s yeast 20 g, soya flour 10 g, malt extract 50 g, nipagin 3 g (dissolved in 70% ethanol), propionic acid 3 g) poured into 90 mm Petri dishes (Fisherbrand™ Polystyrene; Fisher Scientific, Loughborough, UK). Adults were held in cages (32.5 cm × 32.5 cm × 32.5 cm; Bugdorm, MegaViewScience, Taichung, Taiwan), with an average population size of approximately 4000–6000 and stored in climate chambers at 23 ± 3 °C, 65 ± 5% relative humidity, and a photoperiod of 16 h light:8 h dark (L:D 16:8) at BigSis (Reading, UK). The founders of the colony were collected in October 2021. By the time the laboratory experiments began, in January 2022, the colonies had gone through 4 to 8 generations in laboratory conditions. Prior to the start of field trials in May 2023, the colonies had been maintained in the laboratory for approximately 34 to 38 generations.

Male *D. suzukii* were sterilised using BigSis proprietary X-ray dose and technology. Adult *D. suzukii* were collected within 24 h of emergence, manually sex-sorted under CO_2_ anaesthesia and marked with fluorescent powder (0.08 g per 1000 flies; S-2800 Series Water Based Acrylic Paint, BioQuip Products, Inc., Compton, CA, USA) as described in Clymans et al. [27]. All adult *D. suzukii* (marked irradiated males, non-irradiated males and females) were held separately in vials (flat-bottom polypropylene tubes; 152 mm length × 26 mm diameter) with sugar-agar (2% *w*/*v* agar, 5% *w*/*v* sucrose, 0.4% *v*/*v* acetic acid) for at least 2 days prior to experiments and/or field releases (see Section 2.2) to allow flies to recover from CO_2_ exposure and to ensure sexual maturity and no prior mating [28]. Adult *D. suzukii* were approximately 3 to 5 days old at the time of the experiment or field release.

#### 2.1.1. Flight Performance

The impact of sterilisation on the flight performance of male *D. suzukii* was assessed in a wind tunnel (internal dimension: L 190 cm × W 67 cm × H 65 cm, carbon filtered; Airclean Ltd., Marden, Kent, UK) using irradiated and non-irradiated (control) males. A standard bucket trap (red base/transparent lid; Suzukii Trap; Russell IPM, Deeside, UK) baited with 200 mL of a commercial lure (RIGA AG, Ellikon an der Thur, Switzerland) separated by a metal woven mesh (1 mm × 1 mm), to prevent flies contacting with the lure, was used to attract the flies. Each replicate (n = 8) used 100 flies of each treatment marked with an identifying colour of fluorescent powder. Both irradiated and control males were placed downwind of the liquid bait in vials with cotton wool bungs and given an acclimatisation period of 15 min in the wind tunnel, after which the cotton wool bungs were removed, and all males were tapped onto a Petri dish on the base of the wind tunnel.

Experiments ran for 180 min with the traps replaced every 30 min. Flies that had arrived at the trap during the 30 min intervals, including those on the outside of the trap, were removed using a laboratory suction pump (Dymax 14; Charles Austen Pumps Ltd., Byfleet, UK). Males were identified by colour using an ultraviolet (UV; light λ = 365 mm) torch to differentiate between irradiated and non-irradiated males. All replicates were conducted at 23 ± 3 °C and 65 ± 5% relative humidity. The wind speed in the wind tunnel was set at 0.3 m/s [29] and confirmed with an anemometer (TA400, Airflow Developments Ltd., High Wycombe, UK) before the start of each experiment.

#### 2.1.2. Mating Competitiveness

Under laboratory conditions, *D. suzukii* mating activity is highest during the first 3 h of the photophase and the majority mate within the first 30 min [30]. Therefore, experimental set-up was performed under a red light (dark condition for the flies), at the end of the scotophase. Irradiated and non-irradiated (control) male *D. suzukii* were marked with different colours of fluorescent powder as described above. They were then tapped into a Bugdorm cage (17.5 cm × 17.5 cm × 17.5 cm; Bugdorm, MegaViewScience, Taichung, Taiwan), followed by unmated females. A total of 100 flies was introduced per cage at a ratio of 2:2:1, i.e., 40 irradiated males, 40 non-irradiated males and 20 unmated females. Standard LED lights (output: 23 W, daylight white) were turned on approximately 1 min after introducing the females.

For each replicate (n = 8), adult flies were observed for 180 min. Mating couples were captured in empty vials and removed from the cages as soon as they initiated mating. The start and end time of copulation were recorded, and a UV torch was used to identify the male as irradiated or non-irradiated as above. Females were transferred into a vial containing a cornmeal media to lay eggs for 24 h at 23 ± 3 °C and 65 ± 5% relative humidity. After removing the female from the vial, the number of eggs were counted, and fecundity calculated (No. eggs/female/24 h) using a stereo microscope (Leica MZ8; Leica Microsystems Ltd., Milton Keynes, UK). After ten days of incubation, the number of pupae were counted, and fertility the egg-to-pupae recovery determined (No. pupae/No. eggs). All vials, for oviposition and larval development, were kept in an incubator at 23 ± 3 °C and 65 ± 5% relative humidity, and photoperiod L:D 16:8.

#### 2.1.3. Courtship

To assess the effect of irradiation on the courtship behaviour of *D. suzukii*, single mating pairs of an unmated female and an irradiated or non-irradiated (control) male were transferred to individual Petri dishes (35 × 10 mm, Corning, Inc., Corning, NY, USA) at the end of the scotophase, and recorded for 60 min using EthoVision software (version 16; Noldus Information Technology, Wageningen, The Netherlands) and hardware (Basler acA1300-60gm GigE camera, Computar Lens M0814-MP2 F1.4 f8mm 2/3″; Basler AG, Ahrensburg, Germany). After completion of the experiments, recordings were reviewed, and the courtship behaviour scored using the Manual Scoring function in EthoVision.

Behaviours were classified, according to [30] as (a) orientation, (b) wing-flittering, (c) leg-tapping, and (d) circling. In addition, the female response towards male courtship was differentiated between (e) acceptance/receptiveness and (f) rejection. However, the display of female acceptance behaviour did not necessarily lead to successful mating. A total of 108 pairs (62 non-irradiated couples (control), and 46 irradiated couples) were recorded during courtship and the frequency and duration of behaviours assessed.

### 2.2. Efficacy of SIT in Commercial Raspberry Crops

The suppression studies in commercial raspberry crops grown under open polytunnels were conducted at four sites across Kent, UK in 2023. All sites are owned by the same company with similar *D. suzukii* pressure historically and planted with the same variety (proprietary; Driscoll’s Inc., Oxnard, CA, USA). One of the sites (10.39 ha), was SIT-treated, and divided into three plots, hereafter referred to as early, mid, and late season; according to when the plots were harvested. Each plot was harvested for 5 or 6 weeks between July and September. The sizes of the early, mid, and late season plots were 3.50, 3.51 and 3.48 ha, respectively. Three other sites were used as control sites (early: 6.30 ha, mid: 6.28 ha, and late: 4.64 ha), chosen to align closely with the expected time of raspberry harvest during the season, thus each SIT-treated plot had an associated comparable control site that did not receive releases of sterile male *D. suzukii*. The minimum distances between the early, mid and late SIT-treated and the respective control sites were 750 m, 20.5 km and 350 m, respectively. Mean numbers of wild females at the start of the study were characteristically low. All four sites were managed according to the grower’s commercial standard. The control sites were managed with the grower’s standard insecticide programme and each received one application of spinosad (application rate: 0.2 L/ha) during the harvesting season, on week 30 (early season site), week 32 (mid-season site) and week 36 (late-season site). Additionally, a single spray of flonicamid (application rate: 0.14 kg/ha) in the mid-season control site was applied pre-fruiting (week 22). The SIT-treated site (all three plots) did not receive insecticide applications during the harvesting season; however, a single spray of deltamethrin was applied to the mid-season plot in week 23.

**Figure 1:**
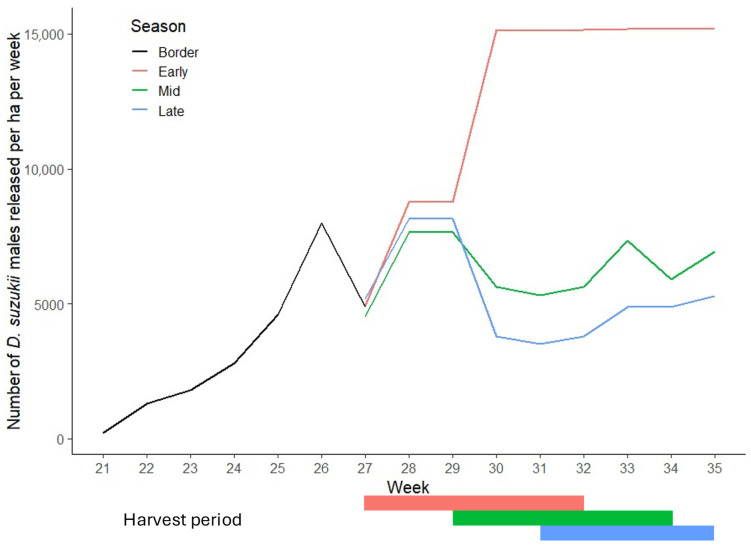
Number of sterile male *Drosophila suzukii* released per ha per week in early (red), mid (green) and late (blue) season harvested plots of SIT-treated commercial raspberry site, by calendar week. In weeks 21 to 27 releases were made only in border areas from week 27 onwards in both, crop and border areas.

Sterile males were released twice a week from calendar week 21 until shortly after the last week of harvesting of the late season plot (calendar week 35). Flies were released into the border areas only during calendar weeks 21 to 26 inclusive; from week 27 onwards, flies were release into both the crop and border areas, with higher concentrations targeted at the plots undergoing harvesting and at areas with comparatively higher numbers of wild *D. suzukii* (Figure 1). The irradiated flies were delivered in vials of 100 and released manually across the plots during late morning, between 09:00 and 11:00.

Correspondingly, monitoring began in May and finished in September. Red sticky traps (210 mm × 100 mm; Russell IPM, Deeside, UK), each with a *D. suzukii* dry lure (SWD, Russell IPM, Deeside, UK) attached, were placed in each site with a density of five evenly spaced traps per ha. Additionally, red sticky traps with attached lures were placed every 80 m around the perimeter of the sites and on pathways within the crop area. The traps were replaced weekly and insects assessed under a light microscope. The number of sterile male, wild male, and wild female *D. suzukii* per trap were recorded. Irradiated males were distinguished by their fluorescent colouring under UV light.

Fruit sampling took place weekly during the commercial harvest period, which was 5 or 6 weeks, for each SIT-treated plot and control site. Eight samples of 100 fruits per plot/site were collected at random from across the respective plots/sites but avoiding fruits from the immediate vicinity of monitoring traps. The samples were sent to the laboratory and incubated for ~48 h at 20 °C to allow time for eggs to hatch and larvae to develop. Larval extraction was performed using the floatation method described in Dreves et al. [31] and the number of larvae recorded.

An ‘a priori’ decision was made for the primary metrics to be fruit infestation and the number of wild female *D. suzukii* per trap during the commercial harvest period, because this is the crucial time to determine the benefit of SIT. Careful efforts were made to select control sites that aligned with the expected harvest time of the corresponding SIT-treated plots; however, harvest time did differ slightly (Table 1). Thus, early and mid-season SIT-treated plots included 6 sampling weeks each, whereas the associated early and mid-season control sites only included 5 sampling weeks. The additional sampling in the SIT-treated plots occurred 1 week after harvest had already stopped in the control site (early season) and 1 week before harvest in the control site started (mid-season). Sampling in the late season plot and site occurred over 5 weeks; however, harvest in the SIT-treated plot started 1 week prior to the control site.

Suppression was quantified by comparing the number of wild adult female *D. suzukii* caught per red sticky traps in the crop of the SIT-treated plots during harvest to the corresponding control sites that did not receive SIT treatment. We also collected data on wild male *D. suzukii*, since these affect the overall *D. suzukii* population, although they do not damage crops.

The total weight (tonnes) of fruit waste per ha during harvest was recorded by the grower. Fruit waste included any fruit removed from the cane by pickers but later rejected during quality control, as well as fruit that had fallen to the ground.

### 2.3. Statistical Analysis

All statistical analyses were performed in R version 4.1.1 (2023.03.30, Build 576) [32].

#### 2.3.1. Quality of Irradiated Sterile Male *D. suzukii*

The flight performance was analysed using a Generalised Linear Mixed Model (GLMM; package ‘glmmTMB’ version 1.1.10 [33]). The model was fitted with a negative binomial/nbinom2 distribution with a ‘log’ link function and the number of *D. suzukii* males captured after 180 min as the response variable. Treatment was included as a fixed factor, with replicate as a random factor. The number of males introduced at the beginning of the experiment was included as an offset. Significance of the main factor (i.e., treatment) was tested using analysis of deviance. Comparison of irradiated vs. control was made using Dunnett’s test from R package ‘emmeans’ version 1.10.7 [34] at the 5% confidence level.

Mating success, egg-to-pupae recovery and mating duration were analysed using Generalised Linear Models (GLM). All models were fit with treatment (i.e., irradiated and control) as a fixed effect. Mating success (i.e., the number of mating pairs) was fit with a Poisson distribution with a ‘log’ link function and the logarithm of the total number of females introduced as offset. The egg-to-pupae recovery of mated females was analysed using GLM with Poisson distribution with a ‘log’ link function and the number of pupae formed as the response variable. The number of eggs laid was included as an offset. The model was fitted using the ‘brglmFit’ method which includes Firth’s correction to account for complete separation within the data [35]. The mating duration was analysed using a linear model with Gaussian distribution and ‘identity’ link function.

Significance of the main factor (i.e., treatment) was tested using analysis of deviance. Comparison of irradiated vs. control was made using Dunnett’s test from R package ‘emmeans’ version 1.10.7 [34] at the 5% confidence level.

For courtship, the frequency and duration of observed mating behaviours and probability of mating success were analysed using GLMs. All models were fitted with the pair number and treatment as fixed effects. The frequency of mating behaviours was fitted with a Poisson distribution with a ‘log’ link function. If the model was overdispersed, it was refitted with a quasi-Poisson or negative binomial distribution to account for overdispersion. The duration of the respective mating behaviours was analysed using a linear model with Gaussian distribution and ‘identity’ link function. The probability of mating success was analysed using a GLM with binomial distribution with a ‘logit’ link function. The model was fit using the ‘brglmFit’ method, as above, which includes Firth’s correction. Significances of the main factors (i.e., pair number and treatment) were tested using analysis of deviance. Comparison between treatments (irradiated vs. control) was made using Dunnett’s test at the 5% confidence level.

#### 2.3.2. Efficacy of SIT in Commercial Raspberry Crops

The population development of *D. suzukii*, namely numbers of wild males and females per trap and numbers of larvae per fruit, was analysed using GLMMs. All models were fitted with a quasi-Poisson/nbinom1 or, if overdispersion was detected, a negative binomial/nbinom2 distribution with a ‘log’ link function and the number of *D. suzukii* females, males, and larvae as the response variable, respectively. Treatment and Sampling Week were included as fixed factors in all models. For analysing adult *D. suzukii* in the cropping area and surrounding hedgerow, Trap nested within Site was included as random factors. The larval infestation was modelled with the sampling point as a random factor. All seasons, i.e., early, mid and late harvest, were analysed separately.

Significances of the main factors (i.e., Treatment and Sampling Week) were tested using analysis of deviance (Type II Wald chi-square). As above, comparison between treatments (SIT vs. control) was made using Dunnett’s test at the 5% confidence level.

Suppression of wild male and female *D. suzukii* was based on the mean across the respective harvest periods. The difference in the SIT-treated plot for trapped wild male and female *D. suzukii* and collected larvae was calculated using the following equation:

% Difference to Control = (Mean SIT-treated − Mean Control)/(Mean Control)

The reduction in fruit waste between the SIT-treated plots and control sites was based on data provided by the grower and calculated using the following equation:

% Difference to Control = ((Waste SIT-treated)/(Total Yield SIT-treated) − (Waste Control)/(Total Yield Control))/((Waste Control)/(Total Yield Control))

However, due to the lack of replicates, the reduction in fruit waste could not be statistically analysed.

## 3. Results

### 3.1. Quality of Irradiated Sterile Male D. suzukii

#### 3.1.1. Flight Performance

Significantly more irradiated compared to non-irradiated (control) *D. suzukii* were recovered, inside or on the outside of the trap in the wind tunnel laboratory test (*Χ*^2^(df = 1) = 11.852, *p* < 0.001). The recapture rates of irradiated and non-irradiated *D. suzukii* males were 76.9% (494 of 648 released flies) and 47.3% (959 of 2025), respectively.

#### 3.1.2. Mating Competitiveness

Unmated female *D. suzukii* showed no significant preference when choosing to mate either irradiated (29.9%) or non-irradiated (26.3%) males (*Χ*^2^(df = 1) = 0.325, *p* = 0.569). Females that mated with fertile non-irradiated males had 80.1% egg-to-pupae recovery whereas females that mated with irradiated males had only 0.7% egg-to-pupae recovery (*Χ*^2^(df = 1) = 55.016, *p* < 0.001). No significant differences were observed in mating duration (*F*(1, 68) = 0.719, *p* = 0.399), which was 18.9 ± 1.44 min when mating with non-irradiated males and 20.6 ± 1.40 min for irradiated males.

#### 3.1.3. Courtship

**Figure 2:**
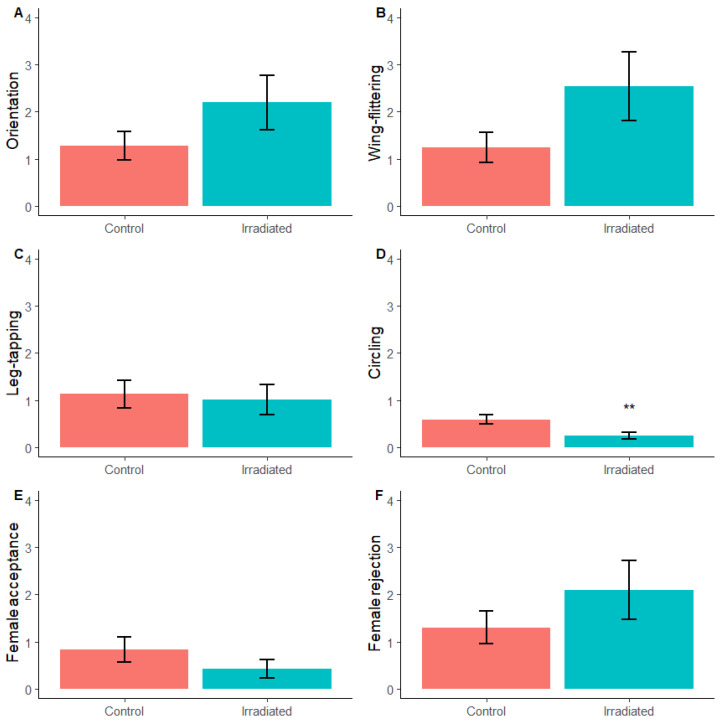
Mean frequency (±SE) of male courtship behaviour (**A**–**D**) and female acceptance (**E**) and rejection (**F**) behaviour expressed during the bioassay. Asterisks indicate significant difference to the non-irradiated control using Dunnett’s test (** <0.01).

A significant reduction in the frequency of circling (observation per hour) was observed for irradiated males (0.244 ± 0.072) compared to non-irradiated males (0.591 ± 0.099; *Χ*^2^(df = 1) = 7.690, *p* = 0.006, Figure 2). The frequency of the other observed courtship behaviours were not significantly different between the two treatments; orientation: *Χ*^2^(df = 1) = 2.367, *p* = 0.124, wing-flittering: *Χ*^2^(df = 1) = 3.503, *p* = 0.061, leg-tapping: *Χ*^2^(df = 1) = 0.351, *p* = 0.782, female acceptance: *Χ*^2^(df = 1) = 8.403, *p* = 0.185 and rejection: *Χ*^2^(df = 1) = 1.479, *p* = 0.224 (Figure 1). Furthermore, no significant differences in cumulative duration were found for any of the courtship behaviours analysed; orientation: *Χ*^2^(df = 1) = 6.130, *p* = 0.224, wing-flittering: *Χ*^2^(df = 1) = 3.170, *p* = 0.539, leg-tapping: *Χ*^2^(df = 1) = 8.989, *p* = 0.074, circling: *Χ*^2^(df = 1) = 1.882, *p* = 0.225).

### 3.2. Efficacy of SIT in Commercial Raspberry Crops

#### 3.2.1. Ratio of Sterile to Wild Male *D. suzukii*

**Figure 3:**
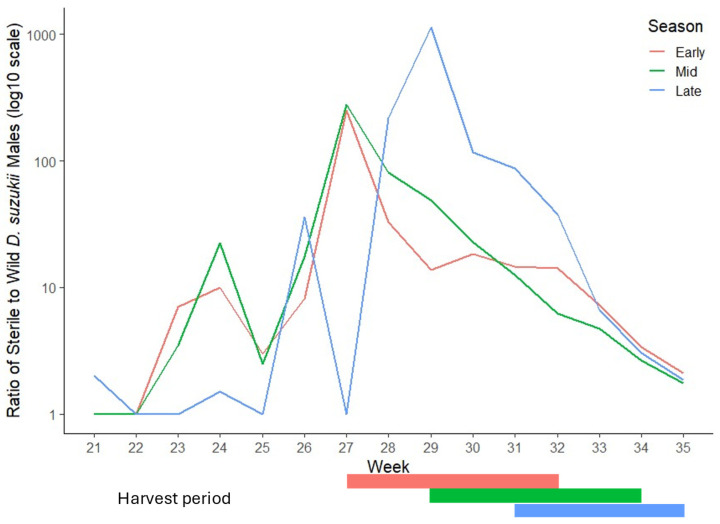
Ratio of sterile to wild male *Drosophila suzukii* in early, mid and late season harvested plots of SIT-treated commercial raspberry site, by calendar week until the end of harvest.

In the early season plot, the ratio of sterile to wild male *D. suzukii* at the onset of harvest (week 27) was 249 sterile males to one wild male, falling to 13 by the end of harvest (week 32) (Figure 3). For the mid-season plot, the ratio declined from 22 in the initial week of harvest (week 29) to 2 by the final week (week 34). In the late-season plot, the ratio fell from 36 during the first week of harvest (week 31) to 0.9 in the last week (week 35).

#### 3.2.2. Suppression of Wild Adult Female *D. suzukii*

**Figure 4:**
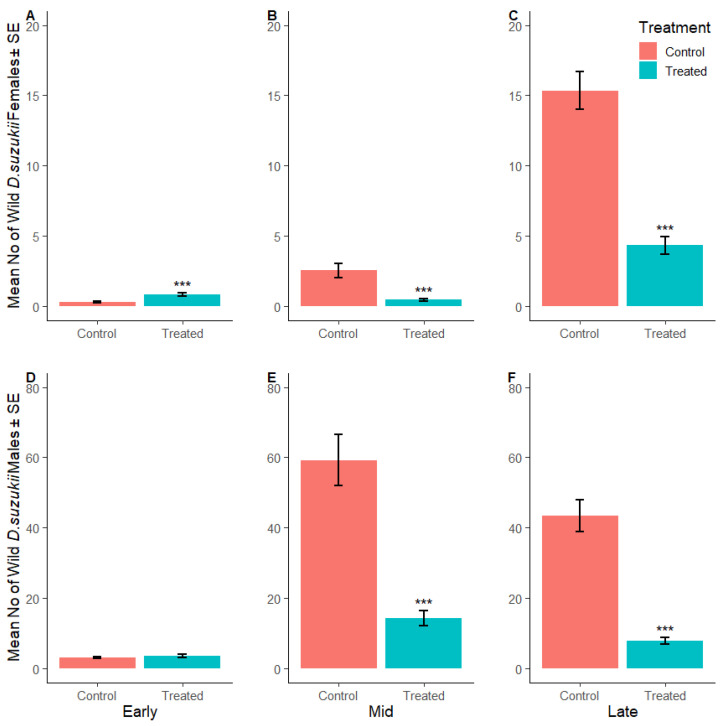
*Drosophila suzukii* in the crop: Mean number of wild female (**A**–**C**) and wild male (**D**–**F**) *D. suzukii* captured per red sticky trap during the early, mid and late harvest periods, respectively, in the control (SIT-untreated; red) and SIT-treated (blue) crop area. Asterisks indicate significant difference to the control using Dunnett’s test (*** <0.001).

The total number of wild female *D. suzukii* caught on red sticky traps in the crop area of the early season SIT-treated plot was 158% higher compared to the corresponding control site during the actual weeks of harvest (Figure 4A). During mid and late season, the number of wild female *D. suzukii* was 82% and 89% lower in the crop area of the SIT-treated plots compared to the control sites, respectively (Figure 4B,C, Table S1).

**Figure 5:**
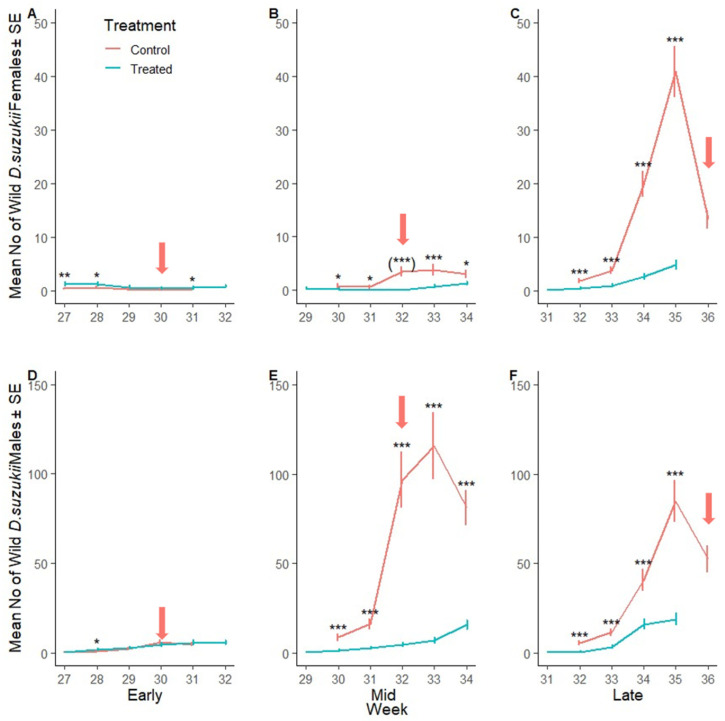
*Drosophila suzukii* in the crop over time: Mean number of wild female (**A**–**C**) and wild male (**D**–**F**) *D. suzukii* captured per red sticky trap over time (week) during the early, mid and late harvest periods, respectively, in the control (SIT-untreated; red line) and SIT-treated (blue line) crop area. Asterisks indicate significant difference to the control for each week using Dunnett’s test (* <0.05, ** <0.01, *** <0.001). (***) indicates statistical significance by complete separation. Arrows indicate insecticide applications in the respective SIT-untreated control sites.

The number of wild male *D. suzukii* in early season was 17% higher in the SIT-treated crop area compared to the control site (Figure 4D). The number of wild male *D. suzukii* became and remained lower in the SIT-treated crop area compared to the control sites for the rest of the trial (Figure 4E,F). Across the mid and late season, the number of wild male *D. suzukii* was lower by 92% and 82%, respectively, in SIT-treated crop areas compared to their control sites. Figure 5A–F show the mean number of wild male and female *D. suzukii* caught on crop area traps in each weak during early, mid and late harvest and when insecticides were applied to the control sites.

**Figure 6:**
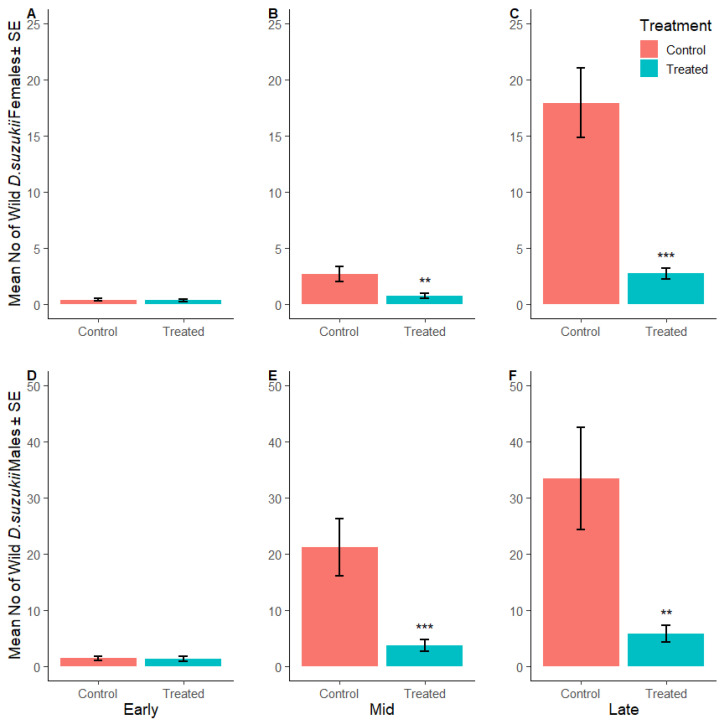
*Drosophila suzukii* in the border: Mean number of wild female (**A**–**C**) and wild male (**D**–**F**) *D. suzukii* captured per red sticky trap during the early, mid and late harvest periods, respectively, in the control (SIT-untreated; red) and SIT-treated (blue) borders. Asterisks indicate significant difference to the control using Dunnett’s test (** <0.01, *** <0.001).

**Figure 7:**
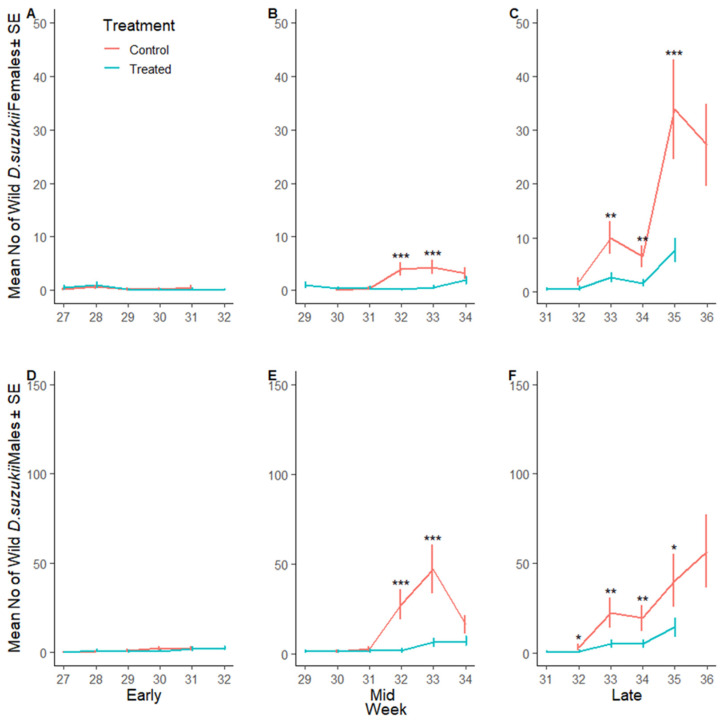
*Drosophila suzukii* in the border over time: Mean number of wild female (**A**–**C**) and wild male (**D**–**F**) *D. suzukii* captured per red sticky trap over time (week) during the early, mid and late harvest periods, respectively, in the control (SIT-untreated; red line) and SIT-treated (blue line) borders. Asterisks indicate significant difference to the control using Dunnett’s test (* <0.05, ** <0.01, *** <0.001).

Border populations of wild female *D. suzukii* were lower in the SIT-treated plots compared to the corresponding control sites in early, mid and late season plots (23%, 79% and 85%, respectively) (Figure 6A–C, Table S2). Similarly, the number of wild male *D. suzukii* was lower in SIT-treated plots in early, mid and late season plots (28%, 86% and 85%, respectively) (Figure 6D–F). Figure 7A–F illustrate the weekly mean count of wild male and female *D. suzukii* captured in traps placed within the surrounding borders during early, mid, and late harvest periods.

#### 3.2.3. Suppression of *D. suzukii* Larvae per Fruit

**Figure 8:**
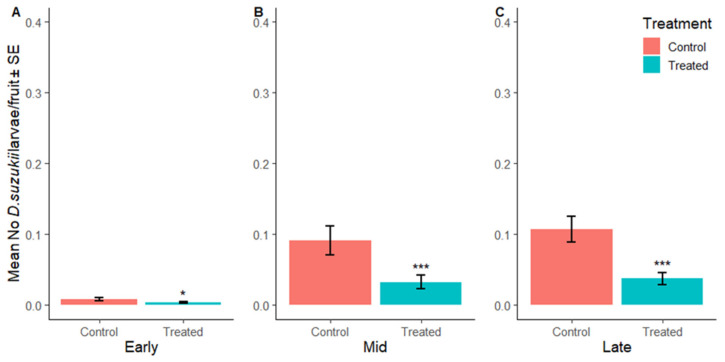
Mean number of larvae per fruit averaged across the respective harvest periods during the early (**A**), mid (**B**) and late (**C**) harvest period in the control (SIT-untreated; red) and SIT-treated (blue) sites. Asterisks indicate significant difference to the control using Dunnett’s test (* <0.05, *** <0.001).

**Figure 9:**
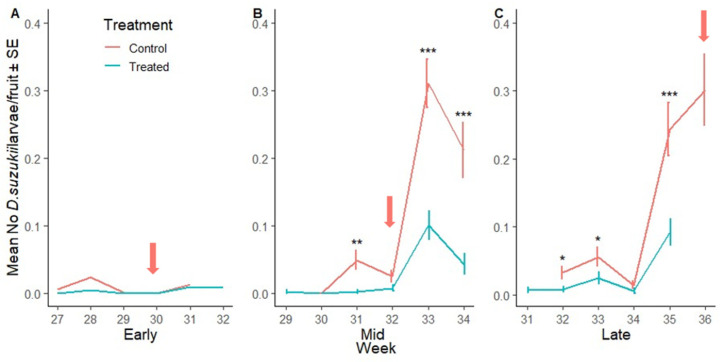
Mean number of larvae per fruit per week during the early (**A**), mid (**B**) and late (**C**) harvest period in the control (SIT-untreated; red line) and SIT-treated (blue line) sites. Asterisks indicate significant difference to the control using Dunnett’s test (* <0.05, ** <0.01, *** <0.001). Arrows indicate insecticide applications in the respective field.

SIT treatment had a significant effect on the number of *D. suzukii* larvae per fruit in early, mid and late season raspberry crops (Table S3 and Figure 8). The overall numbers of larvae per raspberry in the early, mid, and late season were 62%, 79% and 80% lower in the SIT-treated plots compared to their corresponding control sites. Figure 9A–C shows the mean number of larvae extracted from fruit samples in each week during early, mid and late harvest and when insecticides were applied to the control sites.

#### 3.2.4. Reduction in Fruit Waste

Fruit waste (tonnes of unmarketable fruit discarded by pickers during harvest) in the SIT-treated plots was reduced by 40.3%, 26.8%, and 58.4% in the early, mid and late harvesting season plots, compared to the corresponding control sites.

## 4. Discussion

This study demonstrated that irradiated male *D. suzukii* could outcompete wild males for mating and that SIT can successfully control *D. suzukii* in commercial raspberry crops. This positive outcome aligns with our previous study, where we showed that SIT was effective for control of *D. suzukii* in strawberry crops [18]. Raspberry has been reported as more susceptible to *D. suzukii* compared to other hosts including blackberries, blueberries, sweet cherries, table grapes, peaches, and strawberries [25], hence this additional study, on raspberry, provides further evidence that SIT could be used across a range of *D. suzukii* susceptible crops.

The laboratory reared irradiated sterile males were more active than their fertile counterparts in laboratory flight assays, with 29.6% more irradiated males reaching the fruit-based bait during the same period compared to non-irradiated males. The reason for the improved flight performance of the irradiated males is unknown but it could be related to radiation hormesis, where low radiation doses show a beneficial effect to the exposed organism (reviewed in Rix and Culter [36]).

In addition, mating competitiveness and courtship behaviour did not differ between irradiated and non-irradiated males. Females that mated with irradiated males had reduced fertility of over 99% compared to females that mated with non-irradiated males, highlighting the effectiveness of irradiation in suppressing fertility, as shown in other studies [18,19,23,37]. Recently, Lanouette et al. [38] also showed that irradiated males had the same mating capacity as non-irradiated males, copulating with 6.4 and 6.9 females, respectively, in a 24 h period. These results are in line with observations in our mating competitiveness assays despite using different male-female ratios. While Lanouette et al. [38] used a ratio of 1:1:1 (sterile male: fertile male: virgin females), our mating competitiveness assays used a ratio of 2:2:1.

Altogether, these laboratory tests suggest that irradiated males would satisfy the fundamental prerequisite of SIT, which is to disperse to locate wild female *D. suzukii* and then compete for, and mate with wild female *D. suzukii* if released into the environment. Hence, the performance of the irradiated males in this study should ensure the success of any SIT program.

In the SIT-treated commercial raspberry crop, the released sterile males suppressed the build-up of the *D. suzukii* population compared to the untreated crops, which resulted in the reduction in both adult females and larvae in fruits. There was a reduction of 82% and 89% in the number of wild female *D. suzukii* in the mid and late season SIT-treated plots during harvest compared to their respective insecticide-managed sites; no insecticides were applied to the SIT-treated plots. Additionally, the insecticide application did not effectively control *D. suzukii* at the mid-season site (see Figure 5B,E), since *D. suzukii* adults were still abundant a week after this application. This phenomenon has also been observed in previous studies [18]. Other fully replicated studies reported chemical insecticides effective for around 14 days against *D. suzukii* in protected cultivation (polytunnels) [39] and that efficiency can be improved with by adding phagostimulants that enhance uptake [40,41]. *Drosophila suzukii* numbers normally begin to increase again after 2 weeks after application as more *D. suzukii* invade the crop from the surrounding habitat [10,17]. However, with SIT, sterile males can also be released into semi-natural habitats to reduce pest pressure from crop borders (see Figure 5). Border areas are otherwise difficult to manage for *D. suzukii* where cultural management practices are difficult to implement, or insecticide applications are prohibited. Other border management strategies could include the co-release of parasitoids, which would be compatible with SIT as parasitoids target *D. suzukii* larvae and pupae in non-crop hosts [42].

Ultimately, the sole reliance on chemical insecticides is unsustainable in the long term due to the potential of resistance development in *D. suzukii*, and environmental degradation [43,44,45,46,47]. SIT could reduce the reliance on insecticides, helping to secure active ingredients for possible future use.

Importantly for fruit growers, SIT reduced fruit waste, a consequence of fewer *D. suzukii* larvae inside fruit resulting in fewer rejections by the harvest workers [48]. Additionally, fewer females emerging in SIT-treated crops decreases the numbers of eggs laid, and consequently, a reduction in fruit epicarp damage preventing other pests and pathogens degrading fruit through *D. suzukii* oviposition points [12]. Also, the release of irradiated rather than genetically modified males [20,22] is more likely to gain regulatory approval, especially in Europe. The method in this study included sorting males from females, to avoid the release of sterile females which might damage fruit through the oviposition of sterile eggs. Gard et al. [24] released ratios (5:1 and 1:1) of sterile to fertile male and female *D. suzukii* but saw no impact on numbers of infested fruit. It is likely that the release ratio was too low, and released sterile females were able to damage fruits allowing fertile females to lay eggs more easily.

Another area of research involves Incompatible Insect Technique (IIT), which makes use of *Wolbachia* strains to manipulate the insect host reproduction through feminisation, parthenogenesis, male killing and/or cytoplasmic incompatibility [49,50], resulting in a female bias population [51]. To our knowledge, IIT has not yet been developed as an effective management strategy for *D. suzukii*. More recently it was suggested that the combination of SIT and IIT where *D. suzukii* that are infected with *Wolbachia* strains might be irradiated with lower doses compared to those required for SIT alone [52,53]. Our studies demonstrate that radiation dose does not adversely affect male *D. suzukii* mating competitiveness or flight performance, and SIT as applied in the field is effective on its own, as a population suppressant and crop protection strategy.

## 5. Conclusions

Our previous work in commercial strawberries grown under open polytunnels [18] combined with the results presented here on the suppression of *D. suzukii* in commercial raspberries grown under open polytunnels suggest that sterile male *D. suzukii* can induce population suppression in both crops throughout the season.

In conclusion, our study demonstrates the potential of SIT as an effective and sustainable strategy for managing *D. suzukii* populations and reducing fruit infestations in raspberry crops. By dynamically targeting high-pressure areas and employing SIT treatments preventatively, wild *D. suzukii* populations can be suppressed to a commercially acceptable level, with up to 89% reduction in wild adult female *D. suzukii* and up to 80% decrease in larvae per fruit compared to grower’s standard insecticide practice. Furthermore, our findings indicate a substantial decrease in relative fruit waste by up to 58%, highlighting the economic and environmental benefits of SIT implementation. These results offer valuable insights for addressing the ongoing threat posed by *D. suzukii* worldwide.

## Figures and Tables

**Table 1:** Crop harvest periods in the commercial raspberry sites (early, mid, and late season). “X” represents fruit samples and red sticky traps collected during harvest periods, which were included in analysis of larval infestation and population development, respectively.

	Early Season		Mid Season		Late Season	
Calendar Week	SIT-Treated	Control	SIT-Treated	Control	SIT-Treated	Control
27	X	X				
28	X	X				
29	X	X	X			
30	X	X	X	X		
31	X	X	X	X	X	
32	X		X	X	X	X
33			X	X	X	X
34			X	X	X	X
35					X	X
36						X

## Data Availability

Supporting population data of *D. suzukii* counts is available upon request from the first and last authors. Some data, e.g., radiation dose, is the Intellectual Property of BigSis and cannot be disclosed. Some data, e.g., tons of fruit waste per hectare, are confidential to the trial site and cannot be provided.

## Supplementary Materials

The following supporting information can be downloaded at: https://www.mdpi.com/article/10.3390/insects16080791/s1, Table S1. Summary of test statistics for the Type II Wald Chi-square tests analysing the interaction between wild female and wild male *Drosophila suzukii* infestation, SIT treatment and time of the year (week) in the cropping area. Asterisks indicate significant effects of the respective factor or interaction (* <0.05, ** <0.01, *** <0.001) at α = 0.05. Table S2. Summary of test statistics for the Type II Wald Chi-square tests analysing the interaction between wild female and wild male Drosophila suzukii infestation, SIT treatment and time of the year (week) in the border. Asterisks indicate significant effects of the respective factor or interaction (* <0.05, ** <0.01, *** <0.001) at α = 0.05. Table S3. Summary of test statistics for the Type II Wald Chi-square tests analysing the interaction between Drosophila suzukii larval infestation, SIT treatment and time of the year (week) in raspberries. Asterisks indicate significant effects of the respective factor or interaction (* <0.05, ** <0.01, *** <0.001) at α = 0.05.

## Abbreviations

The following abbreviations are used in this manuscript:

SIT	Sterile Insect Technique
AI	Artificial Intelligence
GLMM	Generalised Linear Mixed Model
GLM	Generalised Linear Model
IIT	Incompatible Insect Technique
