# Supplementary material for: Suppression of Spotted Wing Drosophila, Drosophila suzukii (Matsumura), in Raspberry Using the Sterile Insect Technique
Source: Insects. 2025 Jul 31;16(8):791. doi: 10.3390/insects16080791 (PMC12386905; doi:10.3390/insects16080791)
Supplement: Supplementary file 1 [file insects-16-00791-s001.zip › insects-3608974-supplementary.pdf]

Supplementary material

Table S1. Summary of test statistics for the Type II Wald Chi-square tests analysing the interaction between wild female and wild male *Drosophila suzukii* infestation, SIT treatment and time of the year (week) in the cropping area. Asterisks indicate significant effects of the respective factor or interaction (* < 0.05, ** < 0.01, *** < 0.001) at $\alpha = 0.05$.

	Response:	Females					Males			
		Chisq	Df	Pr(>Chisq)			Chisq	Df	Pr(>Chisq)	
Early season	Week	23.606	5	< 0.001	***	Week	198.139	5	< 0.001	***
	Treatment	18.329	1	< 0.001	***	Treatment	0.346	1	0.556	
	Week × Treatment	1.101	4	0.894		Week × Treatment	8.494	4	0.075	
Mid season	Week	60.011	5	< 0.001	***	Week	400.351	5	< 0.001	***
	Treatment	18.138	1	< 0.001	***	Treatment	192.136	1	< 0.001	***
	Week × Treatment	5.314	4	0.257		Week × Treatment	44.707	4	< 0.001	***
Late season	Week	490.863	5	< 0.001	***	Week	565.028	5	< 0.001	***
	Treatment	167.372	1	< 0.001	***	Treatment	70.808	1	< 0.001	***
	Week × Treatment	5.431	3	0.143		Week × Treatment	12.096	3	0.007	**

Table S2. Summary of test statistics for the Type II Wald Chi-square tests analysing the interaction between wild female and wild male *Drosophila suzukii* infestation, SIT treatment and time of the year (week) in the border. Asterisks indicate significant effects of the respective factor or interaction (* < 0.05, ** < 0.01, *** < 0.001) at $\alpha = 0.05$.

	Response:	Females					Males			
		Chisq	Df	Pr(>Chisq)			Chisq	Df	Pr(>Chisq)	
Early season	Week	14.884	5	0.011	*	Week	46.820	8	< 0.001	***
	Treatment	0.029	1	0.864		Treatment	9.009	4	0.061	
	Week × Treatment	6.138	4	0.189		Week × Treatment	8.735	4	0.068	
Mid season	Week	45.333	5	< 0.001	***	Week	182.357	5	< 0.001	***
	Treatment	6.641	1	0.010	**	Treatment	12.232	1	< 0.001	***
	Week × Treatment	26.478	4	< 0.001	***	Week × Treatment	41.395	4	< 0.001	***
Late season	Week	139.315	5	< 0.001	***	Week	164.317	5	< 0.001	***
	Treatment	19.869	1	< 0.001	***	Treatment	8.700	1	0.003	**
	Week × Treatment	0.639	3	0.888		Week × Treatment	1.162	3	0.762	

Table S3. Summary of test statistics for the Type II Wald Chi-square tests analysing the interaction between *Drosophila suzukii* larval infestation, SIT treatment and time of the year (week) in raspberries. Asterisks indicate significant effects of the respective factor or interaction (* < 0.05, ** < 0.01, *** < 0.001) at $\alpha = 0.05$.

	Response:	Larvae			
		Chisq	Df	Pr(>Chisq)	
Early season	Week	67.568	5	< 0.001	***
	Treatment	6.339	1	0.012	*
	Week × Treatment	7.166	4	0.127	
Mid season	Week	109.297	5	< 0.001	***
	Treatment	29.456	1	< 0.001	***
	Week × Treatment	5.359	4	0.252	
Late season	Week	101.680	5	< 0.001	***
	Treatment	13.036	1	< 0.001	***
	Week × Treatment	0.980	3	0.806	
